# Identification of UBE2T as an independent prognostic biomarker for gallbladder cancer

**DOI:** 10.3892/ol.2020.12230

**Published:** 2020-10-15

**Authors:** Xuan Zhu, Tao Li, Xing Niu, Lijie Chen, Chunlin Ge

Oncol Lett 20: Article number 44, 2020; DOI: doi.org/10.3892/ol.2020.11903

Owing to an error that occurred during the production process, a couple of the affiliations for the above paper were published either incorrectly or in an incomplete manner. For the second address affiliation, “Shenyang, Liaoning 110001” should have appeared as “Anshan, Liaoning 114011”; furthermore, for the third affiliation, “Liaoning Health Industry Group” should also have been included as a part of the address. Therefore, the authors’ affiliation information for this paper should have appeared as follows (changes are highlighted in bold):

XUAN ZHU^1,2^, TAO LI^3^, XING NIU^4^, LIJIE CHEN^5^ and CHUNLIN GE^1^

^1^Department of General Surgery, First Affiliated Hospital of China Medical University, Shenyang, Liaoning 110001; ^2^Department of General Surgery, Anshan Hospital, First Affiliated Hospital of China Medical University, **Anshan, Liaoning 114011**; ^3^Department of General Surgery, Fukuang General Hospital, **Liaoning Health Industry Group**, Fushun, Liaoning 113008; ^4^Department of Second Clinical College, Shengjing Hospital Affiliated to China Medical University, Shenyang, Liaoning 110004; ^5^Department of Third Clinical College, China Medical University, Shenyang, Liaoning 110122, P.R. China

The authors regret their oversight in this regard, and apologize for any inconvenience caused.

